# Arginine methylation augments Sbp1 function in translation repression and decapping

**DOI:** 10.1111/febs.15057

**Published:** 2019-09-23

**Authors:** Nupur Bhatter, Raju Roy, Shanaya Shah, Sneha P. Sastry, Sabnam Parbin, Rajan Iyappan, Siddharth Kankaria, Purusharth I. Rajyaguru

**Affiliations:** ^1^ Department of Biochemistry Indian Institute of Science Bangalore India; ^2^ University of California Davis CA USA; ^3^ IAPG Academy of Science Libechov Czech Republic

**Keywords:** decapping, eIF4G, RGG‐motif proteins, RNA granules, Sbp1, translation repression

## Abstract

The fate of messenger RNA in cytoplasm plays a crucial role in various cellular processes. However, the mechanisms that decide whether mRNA will be translated, degraded or stored remain unclear. Single stranded nucleic acid binding protein (Sbp1), an Arginine‐Glycine‐Glycine (RGG‐motif) protein, is known to promote transition of mRNA into a repressed state by binding eukaryotic translation initiation factor 4G1 (eIF4G1) and to promote mRNA decapping, perhaps by modulation of Dcp1/2 activity. Sbp1 is known to be methylated on arginine residues in RGG‐motif; however, the functional relevance of this modification *in vivo* remains unknown. Here, we report that Sbp1 is arginine‐methylated in an hnRNP methyl transferase (Hmt1)‐dependent manner and that methylation is enhanced upon glucose deprivation. Characterization of an arginine‐methylation‐defective (AMD) mutant provided evidence that methylation affects Sbp1 function *in vivo*. The AMD mutant is compromised in causing growth defect upon overexpression, and the mutant is defective in both localizing to and inducing granule formation. Importantly, the Sbp1‐eIF4G1 interaction is compromised both for the AMD mutant and in the absence of Hmt1. Upon overexpression, wild‐type Sbp1 increases localization of another RGG motif containing protein, Scd6 (suppressor of clathrin deficiency) to granules; however, this property of Sbp1 is compromised in the AMD mutant and in the absence of Hmt1, indicating that Sbp1 repression activity could involve other RGG‐motif translation repressors. Additionally, the AMD mutant fails to increase localization of the decapping activator DEAD box helicase homolog to foci and fails to rescue the decapping defect of a *dcp1‐2Δski8* strain, highlighting the role of Sbp1 methylation in decapping. Taken together, these results suggest that arginine methylation modulates Sbp1 role in mRNA fate determination.

## Graphical abstract

The Arginine‐Glycine‐Glycine motif translation repressor protein single stranded nucleic acid binding protein (Sbp1) is methylated on an arginine residue by hnRNP methyl transferase. Here, the role of arginine methylation in modulating the function of Sbp1 is explored in yeast. Upon glucose starvation, there is enhanced mono‐methylation of Sbp1. Arginine methylation positively regulates the interaction of Sbp1 with eukaryotic translation initiation factor 4G1, which in turn promotes its translation repression activity. This modification also promotes the role of Sbp1 in mRNA decapping.

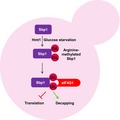

**Abbreviations**
Dhh1: DEAD box helicase homolog
Edc3: enhancer of mRNA decapping
eIF4G1: eukaryotic translation initiation factor 4G1
GST: Glutathione *S*‐transferase
Hmt1: hnRNP methyl transferase
Pab1: poly A binding protein
P‐bodies: processing bodies
PD: Pull down
RGG‐motif: Arginine-Glycine-Glycine
RRM: RNA recognition motif
Sbp1: single stranded nucleic acid binding protein
Scd6: suppressor of clathrin deficiency

## Introduction

Regulation of mRNA translation and decapping plays a crucial role in mRNA fate determination. Since translation initiation and decapping are inter‐related, factors that affect translation also modulate decapping and vice‐versa 1. Translation repressors are proteins that negatively regulate mRNA translation. Of the few repressors whose repression mechanisms have been characterized (eIF4E‐binding protein, CUP, DEAD box helicase homolog (Dhh1), Ded1 2, 3, 4, 5, most seem to target steps during translation initiation. Single stranded nucleic acid binding protein (Sbp1) was recently identified as one of the Arginine‐Glycine‐Glycine (RGG‐motif) containing proteins that repress translation by binding eukaryotic translation initiation factor 4G1 (eIF4G1) through its RGG‐motif 6. Besides the recently reported role of RGG‐motif containing proteins in translation control, this group of proteins are known to be involved in various important biological functions such as transcription, DNA damage and repair, synaptic plasticity, viral pathogenesis and T‐cell signaling 7.

Sbp1 (earlier known as Single stranded DNA/RNA binding protein 1, Ssb1) is an RGG‐motif containing RNA binding protein implicated in mRNA decay and translation repression 8. RNA binding domains, RNA recognition motif (RRM)1 and RRM2, flank the central RGG‐motif of Sbp1 through which Sbp1 binds eIF4G1 to repress translation. The RGG‐motif of Sbp1 is sufficient to bind eIF4G1 and repress translation, *in vitro*. Sbp1 has been also shown to act as a decapping activator 8 since it rescues the decapping defect of *dcp1‐2Δski8* and *dcp2‐7Δski3* strains. The mechanism by which Sbp1 affects translation and decapping is unclear. It is possible that Sbp1 directly modulates decapping activity by binding decapping complex or mRNA or both. Consistent with its role in repression and decapping, Sbp1 localizes to RNA granules 9 akin to other translation repressors such as FMRP, CUP, Ded1 and Pat1 10, 11, 12, 13. Co‐localization of Sbp1 foci with enhancer of mRNA decapping (Edc3) foci (PB marker) is slightly more than with Pub1 (SG marker) foci 9 but the significance of this observation is unclear. Sbp1 has been identified to bind a subset of mRNAs with certain positional specificity (5′ UTR region) but surprisingly no sequence specificity has been reported 9. It is possible that recruitment to target mRNAs is preceded and guided by binding to eIF4G1 9.

Single stranded nucleic acid binding protein has similar domain organization to mammalian protein Nucleolin 8 which is involved in chromatin remodeling, transcription, RNA processing and nucleo‐cytoplasmic shuttling. Nucleolin is overexpressed in cancer and has oncogenic effects. Interestingly, Nucleolin is one of the most abundant arginine methylated nucleolar protein 14. However, the impact of methylation on its role in translation and mRNA decay remains unclear.

Eukaryotic initiation factor 4G1 is a conserved translation initiation factor that provides a scaffold for recruitment of initiation factors such as poly A binding protein (Pab1), eIF4E and eIF4A. The interaction of a subset of RGG‐motif containing repressors with eIF4G1 raises the issue of functional significance of several RGG‐motif proteins binding to eIF4G1. It is possible that each RGG‐eIF4G complex affects a specific subset of mRNAs 7 that is identified through the RNA‐binding domains fused to RGG‐motifs of repressors. It is also possible that two or more repressors could affect common mRNA targets. In fact, mRNAs cross‐linked to Sbp1 are most related to those interacting with Dhh1 9 however the functional relevance of this overlap needs to be addressed. Understanding the regulation of RGG‐eIF4G interaction would help address the physiological relevance of individual RGG‐eIF4G mRNPs. Since RGG‐motifs can be sites of arginine methylation it is possible that methylation could regulate RGG‐eIF4G1 interaction. Interestingly, Sbp1 gets arginine methylated *in vivo* 15 but the molecular contribution of this modification towards Sbp1 role in repression or decapping is unknown.

Arginine methylation has emerged as an important posttranslational modification especially of RNA‐binding proteins since this group of proteins is the largest representative of arginine methylated proteins 16. Arginine methylation affects protein‐protein and protein‐RNA interactions in both positive and negative manner 16, 17. PRMT1 and its homolog hnRNP methyl transferase (Hmt1) are the major methyltransferases in higher metazoans and yeast. Interestingly self‐association of PRMT1 and Hmt1 is crucial for its activity 18, 19. Hmt1 catalyzes mono‐methylation and asymmetric dimethylation of arginines. Numerous substrates have been identified for PRMT1/Hmt1. Although RGG‐motifs are preferred substrate modification sites of these enzymes 20 the basis for substrate specificity remains obscure. Identifying physiological cues that lead to condition‐specific methylation patterns will be a key to understand the substrate specificity mechanisms. We hypothesized that arginine methylation could affect Sbp1 function. We report here that Sbp1 is methylated in Hmt1–dependent manner *in vivo* and methylation promotes the role of Sbp1 in repression and decapping.

## Results

### Sbp1 is arginine methylated in Hmt1‐dependent manner *in vivo*

**Figure 1:**
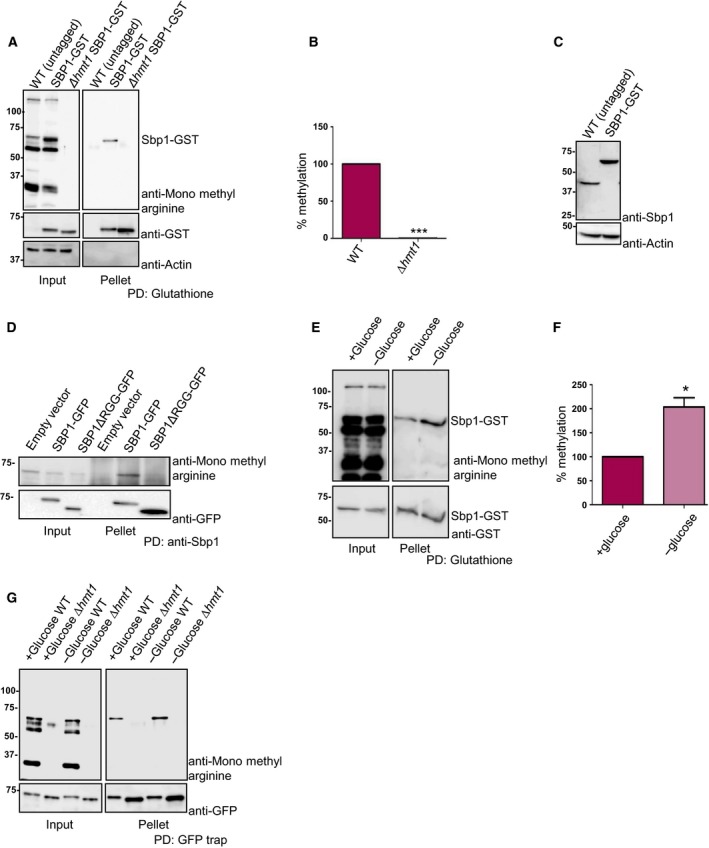
Sbp1 is arginine methylated in Hmt1 and RGG‐motif dependent manner. (A) GST tag was integrated downstream of SBP1 gene in WT and Δhmt1 strain to create a strain expressing SBP1‐GST in WT and Δhmt1 background. Glutathione pull‐downs were performed from these strains followed by probing with anti‐monomethyl arginine specific antibody followed by stripping and probing with anti‐GST (*n* = 3). In the ‘input’ lane, the band that cross‐reacts with mono‐methyl arginine specific antibody and runs at the same size as Sbp1‐GST represents not just Sbp1‐GST but other arginine methylated proteins of similar molecular weight. However in pellet lane such a band is absent in WT (untagged) and only present in SBP1‐GST lane indicating that we are specifically pulling down Sbp1‐GST protein. (B) Quantitation of bands as observed in pellet lanes of (A) as mean SEM with *P*‐value of < 0.0001 calculated using two‐tailed paired *t*‐test. (C) SBP1‐GST strain and wild‐type BY4741 (untagged) strain lysate were probed with anti‐Sbp1 antibody to see relative levels of tagged vs. untagged Sbp1 protein *in vivo*. Actin served as a loading control. (D) SBP1‐GFP was cloned in pRS315 under its own promoter. SBP1ΔRGG was created by deleting 125–167 amino acids by site‐directed mutagenesis. Sbp1 and mutant were pulled down using anti‐Sbp1 antibody raised in rabbit. Pull‐down samples were probed with anti‐mono methyl arginine specific antibody. The same blot was stripped and probed with GFP antibody (Biolegend). (E) Strain expressing endogenously tagged SBP1‐GST was grown until 0.5–0.55 OD_600_ followed by 10 min of glucose deprivation. Glutathione pull‐downs were performed followed by probing with anti‐monomethyl arginine specific antibody. The blot was subsequently stripped and probed with anti‐GST antibody. Increased signal intensity in ‘input’ of 1E as compared to 1A is due to difference in total protein being taken for glutathione pull down. (F) Represents quantitation of bands as mean SEM (*n* = 4) performed as described in E (*P*‐value = 0.0125, calculated using two‐tailed paired *t*‐test, *denotes statistical significance. (G) Sbp1‐GFP cloned in pRS315 plasmid under its own promoter was transformed in BY4741 as well as ∆hmt1 and glucose starved as done in (E). Blot was first probed with mono‐methyl arginine antibody, followed by stripping and probing with anti‐GFP.

Single stranded nucleic acid binding protein is known to be arginine methylated, however the enzyme responsible for methylation *in vivo* has not been unequivocally identified. Although Hmt1, the predominant methyltransferase in yeast, has been reported to methylate Sbp1 *in vitro* 21 a systematic analysis of the methyltransferase(s) required for Sbp1 methylation *in vivo* remains to be done. It was reported earlier that a protein with molecular mass similar to Sbp1 (tentatively identified as Sbp1) showed differential mobility in absence of Hmt1 22. To test if Hmt1 is required to methylate Sbp1 *in vivo*, we pulled down Sbp1‐Glutathione *S*‐transferase (GST) from wild type and *Δhmt1* strains. As a control, wild‐type untagged strain of BY4741 was used. Previous reports indicate that Sbp1 tagged with GST and GFP interacts with other binding partners and localize to RNA granules respectively indicating that such tags do not alter its activity 8, 9, 23. The pull‐downs were probed with mono‐methyl arginine specific antibody (CST, cat#8711) followed by stripping and re‐probing the same blot with anti‐GST antibody (CST, cat#2624). The results indicate (Fig. 1A) that Sbp1 gets arginine methylated in yeast cells as reported earlier 15, 21. Importantly the mono‐methyl arginine‐specific signal is absent when Sbp1‐GST is pulled down from *Δhmt1* strain (Fig. 1A,B). Figure 1C compares the expression level of Sbp1 in strain where it is genomically tagged to GST with untagged Sbp1 from wild type cells. Our result (Fig. 1C) indicates that Sbp1 levels are slightly increased upon GST tagging at its C‐terminus. We further observe that the RGG‐motif deleted mutant of Sbp1 was highly defective in methylation (Fig. 1D) as compared to wild type Sbp1. We conclude based on above results that Sbp1 is arginine methylated in Hmt1 and RGG‐motif dependent manner in yeast cells.

### Sbp1 methylation increases in response to glucose deprivation stress

Post‐translational modifications often regulate protein function by fine‐tuning its activity via modulating interaction with other proteins and/or nucleic acid. If arginine methylation was indeed a way to regulate Sbp1 activity then we hypothesized that the methylation status of Sbp1 might change in response to certain physiological cue(s). Finding such a physiological cue would be crucial to understanding relevance of Sbp1 methylation. To address this, we compared methylation status of Sbp1‐GST from cells subjected to different conditions (growth phases, glucose deprivation and sodium azide treatment). We observed approximately two‐fold increase in methylation of Sbp1 from glucose‐deprived cells as compared to untreated cells (Fig. 1E,F). Increase in mono‐methylation of Sbp1 upon glucose deprivation was also dependent on Hmt1 as mono‐methylation signal was undetectable in absence of Hmt1 (Fig. 1G). Our result suggests that Sbp1 mono‐methylation increases in response to glucose deprivation, which is known to cause global translation repression. We interpret this observation to hypothesize that methylation could affect its function.

### RGG‐motif plays an important role in rescue of Sbp1 overexpression mediated growth defect

**Figure 2:**
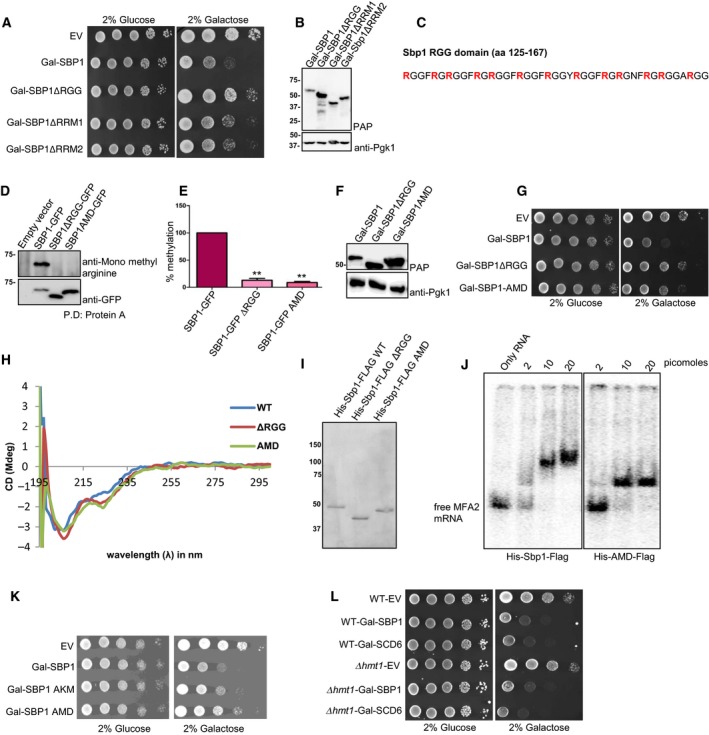
AMD mutant is defective in causing the overexpression mediated growth defect. (A) Deletions of RGG‐motif, RRM1 and RRM2 domains were created in vector expressing galactose inducible SBP1 (BG1805 backbone). Growth assay was performed using BY4741 strains expressing WT and mutants of SBP1 by spotting on SD‐URA plates with 2% glucose or 2% galactose. In general glucose plates were photographed on day 2 whereas galactose plates were photographed on day 3. (B) Galactose inducible WT and Sbp1 mutants were induced in WT yeast cells for 12 h. Lysates were probed with the PAP reagent, which sensitively detects ZZ tag (protein A) present in Sbp1 and its mutants. The same blot was then probed with anti‐Pgk1. Pgk1 served as a loading control. (C) Amino acid sequence of the Sbp1 RGG‐motif (125–167 amino acids) has been shown. Thirteen arginines converted to alanines in the AMD mutant are shown in bold. (D) WT and Sbp1 mutants were expressed under their own promoter and were tagged with GFP at the C‐terminus in WT yeast cells. Anti‐Sbp1 antibody was used to pull‐down Sbp1‐GFP/mutants followed by probing with anti‐mono methyl arginine specific antibody to know the methylation status of Sbp1 mutants (*n* = 3). The same blot was stripped and probed with anti‐GFP antibody. (E) Quantitation of data as mean SEM presented in D. *P*‐value for methylation difference between WT Sbp1 and ∆RGG is 0.0016 while between WT and AMD is 0.0004, calculated using two‐tailed paired *t*‐test. (F) Protein levels of AMD mutant were tested as described in B. (G) Growth assay was performed using strains expressing galactose‐inducible WT and mutants of SBP1 by spotting on SD‐URA plates with 2% glucose or 2% galactose. In general glucose plates were photographed on day 2 whereas galactose plates were photographed on day 3. (H) CD analysis of Sbp1 mutants. Purified wild type and Sbp1 mutants were subjected to CD analysis at 25 °C in PBS buffer without DTT and glycerol. Similar profiles were obtained in all the 5 experiments. (I) CBB stained gel showing proteins used for CD. (J) Sbp1 WT and AMD mutant can bind to MFA2 mRNA. The 100 bp ^32^P‐labeled MFA2 mRNA fragment transcribed from pPIR32 was incubated with indicated concentrations of purified protein in binding buffer (10 mm Tris [pH 8], 50 mm NaCl, 0.05% NP‐40, 6% glycerol, 1 mm DTT supplemented with 1 μg·mL^−1^ BSA and 6.25 ng·mL^−1^ tRNA) at 30 °C for 1 h. The reaction was subjected to vertical native PAGE (4% in 30% acrylamide/bisacrylamide) and run at 100 V for 1 h in TBE buffer (pH 8). The gel was transferred onto a Whatmann 3 mm filter paper and dried for 2 h at 80 °C on a vacuum gel dryer. The dried gel was exposed to phosphor screen and scanned by phosphorimager. (K) Galactose inducible AKM (13 Arginines converted to lysine) of Sbp1 was transformed into wild type yeast cells followed by growth assay as described earlier. (L) Galactose‐inducible Sbp1 and Scd6 were transformed into WT and *Δhmt1* strain. Growth assays were performed using three transformants by spotting the cultures on SD‐URA plates containing either 2% glucose or 2% galactose. In general glucose plates were photographed on day 2 whereas galactose plates were photographed on day 3.

Overexpression of translation repressors such as Dhh1, suppressor of clathrin deficiency (Scd6) and Ded1 leads to growth defect due to global translation repression 2, 3, 24. We hypothesized that translation repressor Sbp1 might also lead to growth defect upon overexpression either due to global translation repression or due to repression of certain key mRNAs required for growth. We observed that cells overexpressing Sbp1 were indeed defective in growth (Fig. 2A). Sbp1 consists of two RRM domains and a central RGG‐motif. We created deletion mutants for each of the domain/motif and tested them in the growth assay. Sbp1 mutants lacking either RRM1 or RRM2 showed a marginal rescue (Fig. 2A). Consistent with the role of RGG‐motif in repression, Sbp1 mutant lacking RGG‐motif compromised the growth defect much more than deletion of RRM1 or RRM2 (Fig. 2A). Protein level analysis of the mutants confirmed that the compromised growth defect was not due to compromised structure that could alter protein levels (Fig. 2B). CD analysis of this mutant indicated that the deletion of RGG‐motif does not significantly alter secondary structure of the mutant protein (Fig. 2H). Our data suggests that the RGG‐motif is important for overexpression mediated growth defect.

RGG‐motif of Sbp1 is important for repressing translation and for arginine methylation. To test if arginine methylation contributed to repression we created an arginine methylation defective mutant (AMD) of Sbp1. A mutant with 13 arginine (all arginines in Sbp1 RGG‐motif shown in bold in Fig. 2C) residues converted to alanine was defective in arginine methylation (Fig. 2D,E). We tested if overexpression of the Sbp1 AMD mutant led to growth defect. We observed that the AMD mutant was compromised in causing the growth defect to a similar extent as the RGG‐deletion mutant (Fig. 2G). The protein levels of the AMD mutant were not compromised as compared to that of the wild type (Fig. 2F). CD analysis of this mutant indicates that arginine to alanine mutations in the AMD mutant does not disrupt secondary structure of the mutant protein (Fig. 2H). To further confirm that the AMD mutant is not compromised in folding, we tested the ability of purified recombinant AMD mutant (Fig. 2J) to bind MFA2 mRNA fragment in electrophoretic mobility shift analysis (EMSA). We observed that purified AMD mutant does bind MFA2 mRNA however the RNA‐protein complex migrates faster than the complex with wild type purified Sbp1. Based on these results we conclude that the AMD mutant is defective in its ability to repress translation.

A mutant with 13 arginines converted to lysine (arginine to lysine mutant – AKM) leads to growth defect upon overexpression however the phenotype is intermediate to wild type and AMD mutant of Sbp1 (Fig. 2K). This could mean that along with arginine methylation, positive charge also contributes to repression activity of Sbp1. Alternatively, compensatory methylation of the substituted lysine residues could support Sbp1 function in this assay. These interesting possibilities will be tested in future studies. Absence of Hmt1 does not affect the ability of Sbp1 to cause growth defect upon overexpression. Scd6 is a related RGG‐motif containing translation repressor whose repression activity is promoted by Hmt1‐mediated arginine methylation 25. Absence of Hmt1 does not affect the growth defect caused by overexpression of either Sbp1 or Scd6 (Fig. 2L) suggesting a role of backup methyltransferase(s) in altering Sbp1/Scd6 function upon overexpression.

### Arginine methylation is important for Sbp1 localization to granules and for binding eIF4G1 *in vivo*

**Figure 3:**
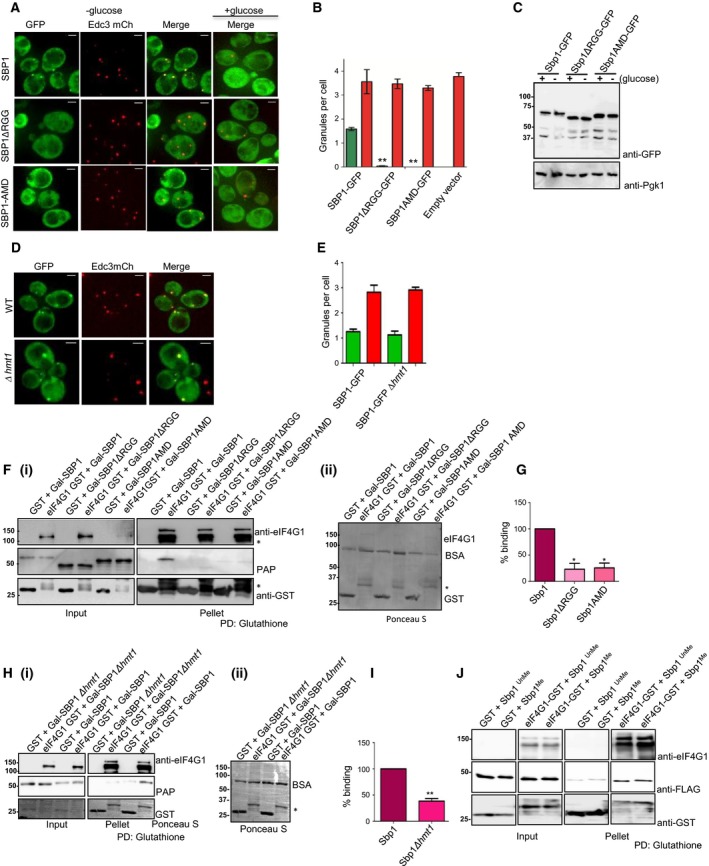
Ability of Sbp1 to get methylated is important for localizing to granules and binding eIF4G1. (A) Plasmids expressing WT Sbp1‐GFP or its mutants were transformed into WT yeast. Cells were grown until 0.4–0.55 OD_600_ followed by 10 min of glucose deprivation. Live cell imaging was performed using DeltaVision microscope (GE) (*n* = 3). (B) Quantitation of data described in (A) using mean SEM. At least 150 cells were counted per experiment. *P* value for difference between WT Sbp1‐GFP and ∆RGG was 0.0026 while WT and AMD was 0.0021, calculated using two‐tailed paired *t*‐test. Green bar indicate Sbp1‐GFP granules, red bar indicate Edc3‐mcherry granules in respective transformants. (C) WT yeast cells expressing Sbp1‐GFP or its mutants were grown as explained in (A) followed by western analysis using anti‐GFP antibody. Pgk1 was used as loading control. On an average 150 cells were counted for each experiment. (D) Sbp1‐GFP granules as observed in WT and *∆hmt1* background. (E) Quant for data described in (D) with *n* = 3 using mean SEM. (F) (i) Purified GST or eIF4G1‐GST was incubated with lysate of yeast cells expressing either wild type Sbp1, Sbp1ΔRGG or AMD mutant. Glutathione pull‐downs were performed and presence of eIF4G1 was confirmed by anti‐eIF4G1 antibody. Sbp1 and its mutants were detected using PAP reagent, which cross‐reacts with protein A tag present in Sbp1. The AMD mutant migrates slightly faster than wild type Sbp1 on SDS/PAGE owing to methylation defect. ‘*’ refers to the degradation products of eIF4G1. ii) Ponceau‐S stained blot for pellet fractions shown (F)(i) showing relative levels of eIF4G1‐GST and control GST proteins. Band running below 75 kDa is BSA that was used in the reaction. (G) Quantitation of data (*n* = 3) presented in (F) using mean SDM. *P*‐value for eIF4G1 binding difference between WT Sbp1 and ∆RGG is 0.0199 while between WT and AMD is 0.0249, calculated using two‐tailed paired *t*‐test. (H)(i) Glutathione pull down of purified eIF4G1‐GST and GST after incubation each of them with BY4741 wild type and *∆hmt1* lysate with over expressed Sbp1 (*n* = 4). (ii) Ponceau‐S stained blot with pellet fractions presented in (H)(i) showing relative levels of eIF4G1 and GST. Full‐length eIF4G1‐GST is not visible because it was too less as compared to control GST protein. A very faint non‐specific band of Sbp1 is present in GST lane as compared to eIF4G1‐GST. (I) Quantitation of PAP band as observed in (H)(i), with a *P* value = 0.0012, calculated using two‐tailed paired *t*‐test. *Refers to statistical significance. (J) Glutathione pull down of eIF4G1‐GST after incubating it with purified *in vitro* methylated Sbp1. Unmethylated Sbp1 was treated the same way as methylated Sbp1 except addition of SAM during the methylation reaction with purified Hmt1. SAM acts as a methyl donor.

RNA granules are conserved mRNP structures that are sites of translation repression 26, 27. Localization to RNA granules [such as processing bodies (P‐bodies) and stress granules] in response to stress is a conserved feature of proteins involved in translation control including many translation repressors such as Sbp1, Scd6, Dhh1 and Ded1 3, 6, 8, 28. Sbp1 co‐localizes with both Edc3 (P‐bodies) and Pub1‐mCherry (stress granules) upon glucose deprivation 9 although the extent of localization was slightly more with Edc3 foci. Since Sbp1 methylation increases upon glucose deprivation, we reasoned that methylation of Sbp1 could affect its function by altering its localization. To test the role of methylation, we created AMD mutant in a plasmid expressing Sbp1‐GFP under its own promoter and observed that the AMD mutant was indeed defective in localizing to RNA granules in response to glucose deprivation (Fig. 3A and green bars in 3B). On the other hand, co‐expressed Edc3‐mCherry showed increased localization to granules upon glucose deprivation in all the cases in comparable manner (Fig. 3A and red bars in 3B) indicating that the inability of the AMD mutant to localize to granules was not due to ineffective glucose starvation stress. We checked the protein levels of AMD mutant under glucose deprivation conditions and noted that it was expressed in manner comparable to the wild type Sbp1 (Fig. 3C). Interestingly localization of Sbp1 upon glucose deprivation is not affected in *Δhmt1* strain (Fig. 3D,E). Based on these results we conclude that AMD mutant is defective in localization to RNA granules.

Single stranded nucleic acid binding protein represses translation by binding eIF4G1. RGG‐deletion mutant of Sbp1 is defective in repressing translation since it is defective in binding eIF4G1 *in vitro* 6. Growth assay as well as granule assay with the AMD mutant indicated that inability to undergo methylation compromised repression activity of Sbp1. We hypothesized that arginine methylation might promote Sbp1‐eIF4G1 interaction directly or indirectly to augment Sbp1 repression activity. We tested the interaction of Sbp1 AMD mutant with eIF4G1 in wild type yeast. We observed that binding of AMD mutant to eIF4G1 was defective in wild type background (Fig. 3F[i,ii],G). Peroxidase anti‐peroxidase (PAP) reagent was used as it detects the protein A tag in Sbp1 with high sensitivity. To confirm that indeed arginine methylation was important for the Sbp1‐eIF4G1 interaction, we tested binding of the two proteins in *Δhmt1* strain. We observed that the Sbp1‐eIF4G1 interaction was compromised in absence of Hmt1 (Fig. 3H[i,ii],I) confirming the role of arginine methylation in promoting the interaction. To test if arginine methylation directly improved the binding of Sbp1 to eIF4G1, recombinant purified Sbp1 from *Escherichia coli* was methylated *in vitro* using purified recombinant Hmt1. Binding of eIF4G1 to unmethylated and hemi‐methylated Sbp1 was compared. However, the results did not reveal significant difference in binding of Sbp1 to eIF4G1 irrespective of its methylation status (Fig. 3J). This observation suggests that the effect of arginine methylation on Sbp1‐eIF4G1 interaction is likely mediated by another factor. Overall our results pertaining to binding of Sbp1 to eIF4G1 *in vivo* indicate that arginine methylation plays an important role in Sbp1‐eIF4G1 interaction.

### AMD mutant is defective in increasing localization of translation repressors and decapping activators such as Scd6 and Dhh1 to foci

**Figure 4:**
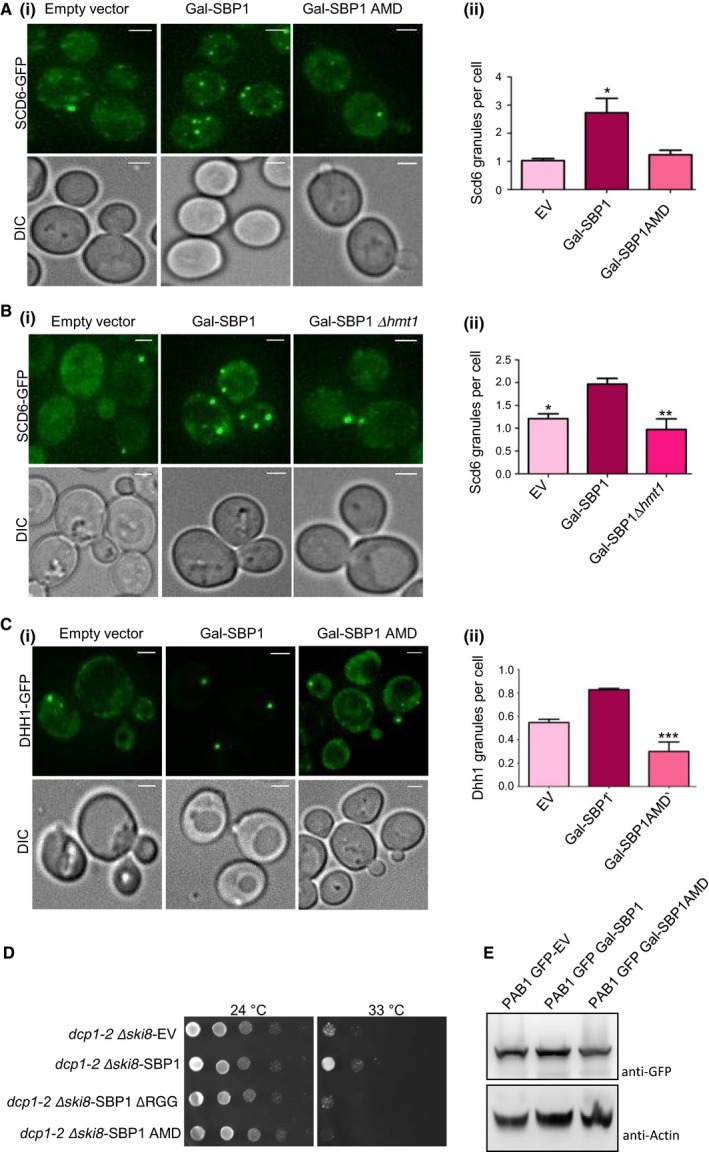
Sbp1 AMD mutant fails to perform the role of Sbp1 in decapping. (A)i) Galactose‐inducible wild type or AMD mutant of Sbp1 was transformed into Scd6‐GFP strain. Cells were grown in SD‐URA with 2% glucose up to 0.3–0.35 OD_600_ followed by growth in 2% galactose containing media for 2 h. (ii) Quantitation of granule count for experiments (*n* = 4) performed in A using mean SEM. At least 150 cells were counted per experiment. *P* value = 0.035, calculated using two‐tailed paired *t*‐test. (B)(i) Ability of Sbp1 to induce Scd6‐GFP granule was compared in WT and *∆hmt1* background. (ii) Quantitation of Scd6‐GFP granule for experiment (*n* = 4) performed in (B)(i) using mean SEM, *P* value = 0.0085, calculated using two‐tailed paired *t*‐test. (C)i) Galactose‐inducible wild type or AMD mutant of Sbp1 was transformed into Dhh1‐GFP strain. Cells were grown in SD‐URA with 2% glucose up to 0.4 OD_600_ followed by growth in 2% galactose containing media for 4 h. (ii) Quantitation of granule count for experiments (*n* = 3) performed in (C)(i). For quantitation a threshold intensity of 1500 was set up for all the images and granules with intensities more than 1500 are counted and plotted. At least 100 cells were counted per experiment using mean SEM. *P*‐value for difference in Dhh1 granule between WT and AMD mutant is =0.0030, calculated using two‐tailed paired *t*‐test. *Denotes statistical significance. (D) Plasmid expressing WT and mutants of Sbp1 were transformed into *dcp1‐2 Δski8* strain. Growth assay was performed by spotting these cultures on SD‐LEU plates and incubating them at 25 °C (permissive temperature) and 33 °C (non‐permissive temperature). Image for 24 °C plate was taken on day 2 while 33 °C plates were imaged on day 4. (E) Pab1‐GFP levels upon overexpression of galactose‐inducible SBP1. Expression of Sbp1 or its mutants in Pab1‐GFP strain was induced for 4 h followed by pelleting and breaking open as described in methods section. Pab1 was detected using anti‐GFP. Actin was used as loading control.

Scd6 and Dhh1 perform dual role of decapping activation and translation repression. Scd6 is an RGG‐motif containing translation repressor that binds eIF4G1 to repress translation 6. We explored if Sbp1 repression activity could involve re‐localization of Scd6 to foci and whether this is dependent on ability of Sbp1 to get methylated. Localization of Scd6‐GFP was assessed upon overexpression of Sbp1. We observed that Scd6 localization to foci increased upon overexpression of Sbp1 but not the AMD mutant (Fig. 4A[i,ii]). Consistent with this observation, localization of Scd6 to foci upon Sbp1 overexpression was defective in absence of Hmt1 (Fig. 4B[i,ii]). We interpret this result to suggest that Sbp1 repression activity perhaps on a subset of mRNAs could involve Scd6. This is an interesting observation and needs to be explored further. Importantly, localization of Scd6 to foci was dependent on ability of Sbp1 to get methylated.

It has been proposed that Sbp1 could contribute to decapping by stimulating decapping activator Dhh1 8, 9 however the mechanism of Sbp1 role in decapping is unclear. Sbp1 overexpression leads to accumulation of Dhh1 in P‐bodies 8, which are sites of mRNA decay leading to the idea that Sbp1 enhances ability of Dhh1 to stimulate decapping. Since arginine methylation of Sbp1 affects its ability to localize to granules, we addressed if overexpression of the AMD mutant could increase Dhh1 localization to foci. We observed that the AMD mutant was defective in increasing localization of Dhh1 to foci (Fig. 4C[i,ii]) indicating that arginine methylation of Sbp1 was required for localization of the decapping activator to foci. This result provides indication that arginine methylation of Sbp1 contributes to its role in decapping.

### Ability of Sbp1 to get methylated is important for suppression of decapping defect

Single stranded nucleic acid binding protein was identified as a multi‐copy suppressor of decapping defect 8, 9 as it rescues the growth defect of *dcp1‐2Δski8* strain. Since arginine methylation was required for localization of decapping activator Dhh1 to granules we decided to test if the Sbp1 AMD mutant could rescue the decapping defect of *dcp1‐2Δski8*. We transformed the WT, RGG domain deletion and AMD mutant of Sbp1 cloned in CEN plasmid (pRS315) under its own promoter, into *dcp1‐2Δski8* strain and checked its ability to grow at non‐permissive temperature. We observed that the WT Sbp1 rescued growth defect at non‐permissive temperatures as reported in literature 8. However, the AMD mutant was defective in rescuing the lethal phenotype (Fig. 4D). This data in combination with Fig. 4A–C provides evidence that the ability of Sbp1 to get methylated likely plays an important role in promoting decapping.

## Discussion

Numerous RGG‐motif containing proteins have been reported to be arginine methylated. Interestingly RNA‐binding proteins form the largest group of arginine‐methylated proteins. However the physiological significance of this modification remains elusive for most of them. In this report, we demonstrate role of arginine methylation in affecting ability of Sbp1 to repress translation. Our conclusion is based on several observations, (a) Endogenous Sbp1 is methylated in RGG‐motif and Hmt1‐dependent manner (Fig. 1A,B,D), (b) Sbp1 mono‐methylation increases in response to glucose deprivation stress (Fig. 1E–G), (c) RGG‐deletion and AMD mutant partially rescues the Sbp1 overexpression growth defect (Fig. 2A,G), (d) AMD mutant is defective in localizing to RNA granules (Fig. 3A,B) and (e) Sbp1‐eIF4G1 interaction *in vivo* is promoted by arginine methylation (Fig. 3F–I).

Our results provide evidence for the role of arginine methylation in affecting decapping. This is based on following observations, (a) the localization of decapping activators Dhh1 and Scd6 to foci upon Sbp1 overexpression is defective in absence of Hmt1 and for the AMD mutant (Fig. 4A–C) and (b) the AMD mutant is defective in rescuing growth defect of *dcp1‐2Δski8* strain (Fig. 4D). The mechanism by which methylation promotes Sbp1 decapping activity remains to be understood and forms an important part of our future plans.

Although it was known in literature that Sbp1 is arginine methylated but the identity of the enzyme that methylates Sbp1 *in vivo* was not clearly established. Result presented in Fig. 1A provide clear idea that arginine methylation of Sbp1 is dependent on Hmt1. This is consistent with the role of Hmt1 as a predominant methyltransferases in yeast. It is also consistent with the role of Hmt1 in methylating RNA‐binding proteins in general including another RGG‐motif protein Scd6 25, 29. Our current results are based on usage of antibody specific for mono‐methyl arginine. Besides using the mono‐methyl arginine antibody, we have also attempted using di‐methylarginine antibodies such as ICP0810, Immunechem 30 and 13522, CST 31 but unlike 8711 (CST) we did not get consistent results both *in vivo* and *in vitro* for Sbp1 with these antibodies hence we are unable to comment on status of dimethylated arginines in Sbp1 in yeast cells. The cross‐reactivity with the yeast total lysate (input) also has been very poor and inconsistent. Although three dimethylated arginines were identified upon *in vitro* methylation of Sbp1 21, the same arginines were observed to be only mono‐methylated *in vivo* 15. The presence of dimethylated arginines in Sbp1 needs to be further experimentally tested. It must be noted that the significance of different kinds of arginine methylation is poorly understood.

Interestingly, absence of Hmt1 does not lead to rescue of Sbp1 overexpression growth defect (Fig. 2L). Residual methylation by backup methyltransferases could mask the growth rescue phenotype. Unlike PRMT1 in mammals, Hmt1 deletion is not lethal in yeast indicating that other methyltransferases(s) perform backup functions in absence of Hmt1. Rmt2, another arginine methyltransferase in yeast that catalyzes N‐δ‐methylation perhaps is not the backup methyltransferase since a strain deleted for both Hmt1 and Rmt2 did not rescue the growth defect (data not shown). Given the above scenario, our current results with AMD mutant serve as a good indicator for the role of arginine methylation in Sbp1 function. One of the important future directions would be identifying the backup methyltransferase of Sbp1. However phenotypes obtained in strains deleted for two or more methyltransferases may not be representative as such phenotypes could be an average consequence of changes in methylation status of several protein substrates *in vivo*.

An interesting finding of our study is that arginine mono methylation of Sbp1 changes in response to glucose deprivation (Fig. 1E–G). A plausible reason for the increased methylation could be change in levels and/or activity of Hmt1 in response to glucose starvation but this has not been reported in literature. Another possibility is that increased access of Hmt1 to Sbp1 in response to glucose stress could contribute to increased methylation, which remains to be experimentally addressed. Importantly increased methylation of Sbp1 upon glucose deprivation affects its function. Sbp1 localizes to granules under this condition and the AMD mutant fails to localize to RNA granules in response to glucose deprivation (Fig. 3A,B) indicating that increased methylation of Sbp1 could promote its localization to granules and translation repression. However, we did not observe any defect in Sbp1 localization to granules upon glucose deprivation in the absence of Hmt1 (Fig. 4C). It will be important to understand methylation (specifically dimethylation) status of Sbp1 during glucose starvation in the absence of Hmt1.

Arginine methylation promotes Sbp1‐eIF4G1 interaction. Both the AMD mutant and the absence of Hmt1 compromises the above interaction (Fig. 3F–I). Since Sbp1 targets eIF4G1 through its RGG‐motif to repress translation, our results indicate that arginine methylation promotes Sbp1 repression activity by augmenting Sbp1‐eIF4G1 interaction. It is notable that recombinant purified Sbp1 binds eIF4G1 in comparable manner irrespective of methylation status (Fig. 3J). We think it is very likely that Sbp1 methylation levels achieved *in vitro* may be insufficient to impact Sbp1‐eIF4G1 interaction. The other strong possibility is that increased interaction between methylated Sbp1 and eIF4G1 observed in Fig. 3F,H is not a direct consequence of Sbp1 methylation. A recent report highlighting the role of RGG‐motif self‐association in regulating repression activity of Scd6 and Sbp1 points in this direction 32. Understanding the basis for increased Sbp1‐eIF4G1 and in general RGG‐eIF4G1 25 interaction upon arginine methylation is an exciting future direction. Based on our results, we propose here that arginine methylation could be a general augmenter of RGG‐motif protein repression activity.

The absence of Hmt1 compromises Sbp1‐eIF4G1 interaction and localization of Scd6 to granules upon Sbp1 overexpression however the growth defect upon Sbp1 overexpression and Sbp1 localization to granules is visibly unaffected by the absence of Hmt1 although defective in presence of the AMD mutant. The differential effect of Hmt1 absence on different Sbp1 functions is intriguing. It raises the possibility that specific kind and/or levels of methylation are required for each Sbp1 function. The residual or compensatory methylation of Sbp1 in absence of Hmt1 is sufficient for enabling overexpression growth defect and for localizing to granules but insufficient to promote eIF4G1 interaction and to increase Scd6 localization to granules upon overexpression. Identifying the methylation status of Sbp1 in absence of Hmt1 and the enzyme(s) responsible for it will be the key to unraveling the basis of above intriguing observations.

Our results indicate that arginine methylation could promote both decapping activation and translation repression by Sbp1. It is possible that upon methylation, the Sbp1‐eIF4G1 interaction could make the cap accessible for decapping enzyme and/or decapping activators leading to activation of decapping of specific sets of mRNA. Testing the consequence of Sbp1‐eIF4G1 interaction on formation of cap‐binding complex will lead to further understanding of this aspect.

Recently Sbp1 was reported to bind Pab1 mRNA in the 5′ UTR region and repress its translation *in vitro* 23. Interestingly this report shows that Sbp1 protein (methylated in *E. coli*) represses translation of luciferase mRNA weaker than unmethylated Sbp1. However, in the same report methylated RGG‐motif of Sbp1 alone was repressing translation in manner comparable to unmethylated RGG‐motif suggesting that the true impact of Sbp1 methylation on Pab1 translation *in vitro* remains to be addressed further. It is also unclear how Sbp1 affects translation of PAB1 mRNA *in vivo*. It is noteworthy that we did not observe decrease in Pab1‐GFP protein levels upon overexpression of Sbp1 (Fig. 4E) which would be expected if Sbp1 represses Pab1 translation. It is possible that repression of PAB1 mRNA by Sbp1 could employ a mechanism different than proposed here and this remains to be addressed in detail. Our results suggest that the eIF4G1 mediated repression mechanism of Sbp1 is positively modulated by arginine methylation.

Understanding the significance of numerous RGG‐motif proteins binding eIF4G will be critical. It is logical to assume that different repressors might undergo changes in methylation status in response to specific conditions like in case of Sbp1. Identifying condition‐specific methylation patterns of other RGG‐motif containing proteins will be an important step towards understanding the physiological cue(s) to which the RGG‐motif containing translation repressors respond. It is likely that alteration of RGG‐motif methylation status will modulate the RGG‐motif bound protein/RNA profile contributing to cue‐specific global changes in translational and mRNA decay output. Such information will provide important insight into repressor‐specific and condition‐specific mRNA fate control in cytoplasm.

## Materials and methods

### Yeast strains and plasmids

All plasmids used in this study are listed in Table S2. Yeast strains used in this study are BY4741 (wild type), *Δhmt1* (Open Biosystems, GE Healthcare, UK), eIF4G1‐GFP, Pab1‐GFP, Sbp1‐GST, Sbp1‐GFP, Scd6‐GFP, Dhh1‐GFP and *dcp1‐2 Δski8* (Table S1). Strains were grown on either standard yeast extract/peptone medium (YP) or synthetic medium (SC) supplemented with the appropriate amino acids and 2% glucose or galactose (when required). All strains except *dcp1‐2 Δski8* were grown at 30 °C. *dcp1‐2 Δski8* was grown at either 24 °C (permissive temperature) or 33 °C (non‐permissive temperature). AMD mutant of Sbp1 was created in four sequential rounds following site‐directed mutagenesis (SDM) protocol using Phusion enzyme (Thermo Scientific, Waltham, MA, USA). The oligonucleotides used for SDM were designed using Quick Agilent site. Arginine to lysine mutant (AKM) of Sbp1 in BG1805 background was synthesized (GenScript, Piscataway, NJ, USA).

### Growth assays

All the strains were patched on appropriate medium and allowed to grow overnight. Next day cells from patches were re‐suspended and OD_600_ was measured. Generally five dilutions were prepared in 96‐well plates starting with OD_600_ 10. In all the growth assays (except in Fig. 4G) equal volume (5 μL) of diluted culture was spotted on both SD‐URA plates with 2% glucose and 2% galactose and incubated at 30 °C. For Fig. 4G, cells were spotted on two SD‐LEU plates with 2% glucose. One of the plates was incubated at 24 °C and the other at 33 °C.

### Protein purification and western blot

Proteins were purified from *E. coli* by batch purification using glutathione sepharose (GE Healthcare, Chicago, IL, USA, catalog no. 17075604) or Ni‐NTA agarose (ThermoFisher Scientific, catalog no. 88222). Cell pellet was resuspended in lysis buffer along with addition of lysozyme (10 μg·mL^−1^), DTT (1 mm), PMSF (2 mm) and Protease Inhibitor Tablet (Roche, Basel, Switzerland), RNase A (1 mg·mL^−1^) for 20 min. Lysate was allowed to bind to beads for 1 h on nutator at 4 °C. Washes were performed on nutator for 10 min at 4°. For His tagged proteins, elution was done with 250 mm of imidazole (SRL cat# 61510) while for GST tagged proteins elution was done using 2 mm reduced glutathione (Amresco cat # 0399‐50G, Solon, OH, Cuyahoga). Purified protein was concentrated and dialyzed into 10 mm Tris–Cl pH7.5, 100 mm NaCl, 20% glycerol and 1 mm DTT in the cold room. Western analysis was performed using anti‐GST (CST, catalog no. 2624; 1 : 1000 dilution), Mono‐methylarginine antibody (CST, catalog # 8711; 1 : 3000 dilution), Di‐methyl antibodies that were tested to detect Sbp1 methylation *in vivo* were CST (cat #13522, 1 : 1000 dilution) and Immunechem (cat #ICP0810, 1 : 500 dilution). PAP [PAP ‐ detects ZZ (protein A) tag fused to Sbp1 expressed under galactose‐inducible promoter with high sensitivity; (Sigma, catalog no. P1291; 1 : 1000 dilution], anti‐GFP (Biolegend, San Diego, CA, USA; 1 : 1000 dilution), anti‐PGK1 (Abcam, Cambridge, MA, USA, catalog no. ab113687; dilution 1 : 1000, St. Louis, MO, USA) and anti‐eIF4G1 (Cocalico Biologicals, PA, USA; 1 : 2000 dilution). Besides being less sensitive than the PAP reagent, anti‐Sbp1 was not used to compare levels of wild type and Sbp1 mutants as this antibody was raised against a full‐length recombinant protein and some of cross‐reactive epitopes could be missing in mutants which may lead to differences in signal intensity for the same amount of wild type and mutant protein. For comparing protein levels of Sbp1 protein and its mutants, cells were lysed using the lysis buffer as mentioned for glutathione pull down and 25 μg of total protein was loaded on an 8% SDS/PAGE gel for western blotting using anti‐GFP antibody. For comparing protein level of genomically tagged Sbp1‐GST and endogenous Sbp1, overnight respective cultures were lysed and 50 μg of total protein was loaded on 10% SDS/PAGE gel, followed by probing with anti‐Sbp1 antibody. To determine total protein levels, the same blot was stripped and probed with anti‐Actin antibody (Abcam, Catalog no. ab8224). For performing western analysis to estimate Pab1 levels upon Sbp1 overexpression, strains were grown in presence of glucose at 30 °C until OD_600_ reached 0.3–0.4. Cells were then pelleted and washed with 2% galactose containing media followed by induction for 4 h. Cells from a 50 mL galactose induced culture were broken open in 100 μL lysis buffer containing 50 mm Tris‐Cl pH7.5, 50 mm NaCl, 2 mm MgCl_2_, 0.1% Triton‐X100, 1 mm β‐Mercaptoethanol, 1× Complete mini‐EDTA‐free tablet (Roche, catalog no. 04693132001) and lysed by vortexing at 4 °C in bead‐beater with glass beads. Unbroken cells and debris were removed by centrifugation at 3381 r.c.f. for 5 min at 4 °C. Twenty microgram of total protein was loaded on SDS/PAGE gel.

### Protein pull‐downs

For testing Sbp1AMD‐eIF4G1 interaction, 100 picomoles of recombinant purified GST or GST‐eIF4G1 was added to lysates expressing wild type or mutant Sbp1 followed by glutathione pull‐downs. Two hundred and fifty millilitre of BY4741 cells transformed with empty vector, WT SBP1 or mutants were grown till 0.45–0.55 OD_600_ in SD‐Ura with 2% glucose. The cultures were pelleted and given two washes with SD‐Ura 2% galactose media. The pellet was suspended in 50 mL of SD Ura‐ with 2% galactose and induced for 6 and 5 h respectively while looking at interaction between AMD‐eIF4G1 in wild type cells and Sbp1‐eIF4G1 in *∆hmt1* cells. Twenty‐five millilitre of induced culture was lysed in 250 μL lysis buffer containing 50 mm Tris–Cl pH 7.5, 50 mm NaCl, 2 mm MgCl_2_, 0.1% Triton‐X100, 1 mm β‐Mercaptoethanol, 1× Complete mini‐EDTA‐free tablet (Roche, catalog no.04693132001) and 2 mm PMSF by vortexing at 4 °C in bead‐beater with glass beads. Unbroken cells and debris were removed by centrifugation at 3381 r.c.f. for 5 min at 4 °C. Three hundred milligram of total protein was used for the pull‐down reactions in 1 mL binding buffer having 50 mm HEPES pH 7.5, 100 mm NaCl, 1% Triton X, 2 mm MgCl_2_, 2 mm MnCl_2_ and 50 μL of glutathione beads. RNase A, DTT and BSA were added to the reaction mixture at a concentration of 1 mg·mL^−1^, 1 mm and 10 mg·mL^−1^ respectively. The reaction mix was nutated at 4 °C for 30 min. Following this, beads were washed twice (10 min each) with binding buffer. After washing, 1× laemmli buffer was added to beads and samples were boiled for 5 min at 100 °C. About 1% of input and 25% of pellet was analyzed by SDS/PAGE followed by western blotting. For *in vitro* interaction studies between Sbp1 and eIF4G1, 100 pm of eIF4G1‐GST and GST were used while 20 pm of Sbp1 (Methylated or Unmethylated) was used. Binding buffer and wash buffer were the same as *in vivo* assay, except that binding was performed for 2 h.

For Protein‐A pull‐down, 5 mL of overnight culture of Sbp1‐GFP and its mutants in SD ‐Leu were pelleted followed by lysis using the lysis buffer described as in for eIF4G‐GST pull‐down. The lysate was incubated with anti‐Sbp1 antibody for 1 h in binding buffer (50 mm Tris pH8, 150 mm NaCl and 0.15% NP40). Twenty microlitre of Protein A Beads (GE Healthcare) were added to each tube followed by incubation for another hour of binding on nutator at 4 °C. The pellet was washed thrice with binding buffer and resuspended in 1× laemmli buffer followed by boiling for 5 min at 100 °C. Fifty percentage of the pellet was loaded on SDS/PAGE gel for western analysis. To prevent antibody bands of anti‐Sbp1 from soaking the secondary antibody, the blot was cut just above 55 kDa after the transfer of the proteins in nitrocellulose membrane. The blot was first probed with mono‐methyl arginine antibody followed by stripping and then probing with anti‐GFP antibody.

For Sbp1‐GST pull‐downs, 100 mL of culture was grown in YEPD till 0.5–0.55 OD_600_. The cultures were divided into equal volume and pelleted only once at 3234 r.c.f. for 10 s at room temperature. The + glucose and – glucose pellets were given one wash at 3234 r.c.f. for 10 s at room temperature with YEPD or YEP respectively. Final resuspension of culture was in 50 mL of YEP or YEPD and allowed to grow for 10 min at 30 °C and 250 r.p.m. Following this, cells were pelleted at room temperature at 3234 r.c.f., 10 s and stored in −80 °C. Cells were lysed using same lysis buffer as mentioned for GST pull‐down. After removing the input sample, the supernatant was nutated for 2 h at 4 °C with 30 μL of glutathione Sepharose‐4B (GE Healthcare) in 500 μL reaction mix with same binding buffer containing 1 mg·mL^−1^ RNase A and 10 mg·mL^−1^ BSA. Beads were washed three times (10 min each) with binding buffer. Twenty microlitre of binding buffer and 1× laemmli buffer was added to beads and analyzed by SDS/PAGE. Five percentage input and 20% of pellet was loaded followed by western blotting.

### GFP trap

Wild type and Hmt1 deletion cells expressing plasmid borne Sbp1‐GFP (under its own promoter) were grown and glucose starved in SD‐leu media like described for Sbp1‐GST strain. Sbp1‐GFP was pulled down using GFP‐trap by following protocol provided by Chromotek (Munich, Germany). Briefly, cell lysate was allowed to bind to beads for 2 h in a nutator at 4 °C. Following binding, the beads were given three washes with binding buffer (as provided in the kit) by settling the beads down in magnetic stand. After three washes, the beads were boiled at 100 °C in 1× laemmli buffer. Five percentage of input and 70% of the pellet was loaded in 8% poly acrylamide gel followed by western blot analysis using mono‐methyl antibody (CST cat # 8711) at a dilution of 1 : 3000 in TBS with 5% skimmed milk. To know the Sbp1‐GFP protein level in the pellet lanes, the same blot was stripped and probed with anti‐GFP (Biolegend catalog no. 902602) antibody.

### Live cell imaging

For glucose starvation experiments with wild type or Hmt1 deletion strains expressing plasmid borne Sbp1‐GFP and Edc3‐mCherry, yeast cultures were grown to OD_600_ of 0.5–0.55 in SD‐Leu‐Ura + 2% glucose media at 30 °C. Glucose starvation was done as described for SBP1‐GST strain. For Dhh1‐GFP and Scd6‐GFP microscopy experiments, yeast cultures were grown to OD_600_ of 0.3–0.35 in the SD‐Ura at 30 °C. Galactose inductions were performed as described above for 2 h for Scd6GFP and 4 h for Dhh1 GFP. After induction, cells were pelleted at 14 100 r.c.f. for 12 s and spotted on coverslips for immediate microscopic examination at room temperature. All images were acquired using Deltavision Elite microscope (GE Healthcare) system running softworx 3.5.1 software (Applied Precision, Bratislava, Slovakia; LLC), using an Olympus 100×, oil‐immersion 1.4 NA objective. Exposure time for GFP channel was 0.2 s for Sbp1‐GFP and Dhh1‐GFP, while 0.25 s for Scd6 GFP. Transmittance was 32% for all GFP strains. Exposure time and transmittance for mCherry channel were 0.3 s and 32% respectively. Images were collected as 512 × 512 pixel files with a CoolSnapHQ camera (Photometrics, Surrey, BC, Canada) using 1 × 1 binning for yeast. All yeast images were deconvolved using standard softworx deconvolution algorithms. ImageJ was used to adjust all images to equal contrast ranges according to the experiment conducted or protein examined. For Sbp1‐GFP experiment on an average, 150 and for Dhh1‐GFP and Scd6‐GFP 100 cells respectively were counted per experiment. For Dhh1‐GFP experiments, softworx 3.5.1 software was used to count the number of granules per cell automatically (an image threshold of 1500 was applied to all the images). Data from three independent experiments was used for quantitation and statistical significance was calculated using paired *t*‐test. Scale bar shown at the top right position of microscopy images corresponds to 2 μm.

### *In vitro* methylation

Purified His‐Sbp1‐FLAG and 1 microgram of His‐Hmt1 were incubated with or without S‐adenosyl methionine (SAM) (NEB) at 30 °C for 12 h in methylation buffer containing 100 mm Tris pH8, 500 mm NaCl, 2 mm EDTA and 1 mm DTT. Proteins were dialyzed in 10 mm Tris pH 7.5, 150 mm NaCl, 1 mm DTT and 20% glycerol for 12 h at 4 °C. Samples were removed and stored in −20 °C. Methylation was confirmed by both mobility shift and western analysis using mono‐methyl antibody (CST catalog no. 8711).

### Circular dichroism

Proteins used for CD were purified using Ni‐NTA protocol and dialysed overnight in PBS buffer without DTT and glycerol. One micro molar of each of the proteins were taken for the assay. The CD spectra were recorded in the range 190–300 nm with a scanning speed of 100 nm·min^−1^ and a response time of 4 s at 25 °C using a Jasco spectrophotometer equipped with a temperature controller (having a quartz cell with 1 cm path length). The experiment was repeated five times. The data was analyzed by using Microsoft Office Excel (Redmond, WA, USA). For plotting graph wavelength was taken from 195 to 300 nm.

### RNA electrophoretic mobility shift assay

pPIR32 was digested with Mnl1 enzyme overnight at 37 °C and digested template was recovered using phenol:chloroform:isoamylalcohol extraction method. One microgram of the cut vector was used as a template to *in vitro* transcribe 100 bp MFA2 mRNA using αP32ATP to body label the RNA. The resultant transcript was purified using RNA purification kit (Thermo cat# AM1908) and stored in −80 °C. To study binding of Sbp1 and its mutant to mRNA, labelled MFA2 RNA was incubated with the protein at 30 °C along with binding buffer at indicated concentration (Fig. 2J). Samples were loaded in a 4% native acrylamide gel followed by drying and exposing to phosphor screen. The screen was developed using Typhoon Phosphor Imager (GE healthcare, Uppsala, Sweden).

## Conflict of interest

The authors declare no conflict of interest.

## Author contributions

PIR conceived the idea. PIR and NB designed the experiments. RR performed DHH1‐GFP microscopy and checked Pab1‐GFP levels upon Sbp1 overexpression. SS created AMD mutant in BG1805 background. SPS performed growth assay for *dcp1‐2∆ski8* with AMD mutant of Sbp1. SP performed EMSA with purified recombinant Sbp1 and AMD mutant provided to her. RI raised anti‐Sbp1 antibody. SK performed growth assay of Sbp1 and Scd6 in *∆hmt1* background. Rest of the experiments were performed by NB. PIR and NB analyzed the data. PIR wrote the first draft of the manuscript. NB and RR helped with editing writing and editing the subsequent drafts.

## Supporting information


**Fig. S1.** Sbp1 overexpression does not lead to decreased 35S incorporation.
**Table S1.** List of strains used in this study.
**Table S2**. List of plasmids used in this study.

## References

[1] Roy D & Rajyaguru PI (2018) Suppressor of clathrin deficiency (Scd6)‐An emerging RGG‐motif translation repressor. Wiley Interdiscip Rev RNA 2018, e1479. 10.1002/wrna.1479 29790275

[2] Coller J & Parker R (2005) General translational repression by activators of mRNA decapping. Cell 122, 875–886. 16179257 10.1016/j.cell.2005.07.012 PMC1853273

[3] Hilliker A , Gao Z , Jankowsky E & Parker R (2011) The DEAD‐box protein Ded1 modulates translation by the formation and resolution of an eIF4F‐mRNA complex. Mol Cell 43, 962–972. 21925384 10.1016/j.molcel.2011.08.008 PMC3268518

[4] Nakamura A , Sato K & Hanyu‐Nakamura K (2004) Drosophila cup is an eIF4E binding protein that associates with Bruno and regulates oskar mRNA translation in oogenesis. Dev Cell 6, 69–78. 14723848 10.1016/s1534-5807(03)00400-3

[5] Sonenberg N & Gingras AC (1998) The mRNA 5′ cap‐binding protein eIF4E and control of cell growth. Curr Opin Cell Biol 10, 268–275. 9561852 10.1016/s0955-0674(98)80150-6

[6] Rajyaguru P , She M & Parker R (2012) Scd6 targets eIF4G to repress translation: RGG motif proteins as a class of eIF4G‐binding proteins. Mol Cell 45, 244–254. 22284680 10.1016/j.molcel.2011.11.026 PMC3277450

[7] Rajyaguru P & Parker R (2012) RGG motif proteins: modulators of mRNA functional states. Cell Cycle 11, 2594–2599. 22767211 10.4161/cc.20716 PMC3873214

[8] Segal SP , Dunckley T & Parker R (2006) Sbp1p affects translational repression and decapping in *Saccharomyces cerevisiae* . Mol Cell Biol 26, 5120–5130. 16782896 10.1128/MCB.01913-05 PMC1489156

[9] Mitchell SF , Jain S , She M & Parker R (2013) Global analysis of yeast mRNPs. Nat Struct Mol Biol 20, 127–133. 23222640 10.1038/nsmb.2468 PMC3537908

[10] Beckham C , Hilliker A , Cziko AM , Noueiry A , Ramaswami M & Parker R (2008) The DEAD‐box RNA helicase Ded1p affects and accumulates in *Saccharomyces cerevisiae* P‐bodies. Mol Biol Cell 19, 984–993. 18162578 10.1091/mbc.E07-09-0954 PMC2262982

[11] Pilkington GR & Parker R (2008) Pat1 contains distinct functional domains that promote P‐body assembly and activation of decapping. Mol Cell Biol 28, 1298–1312. 18086885 10.1128/MCB.00936-07 PMC2258743

[12] Gareau C , Houssin E , Martel D , Coudert L , Mellaoui S , Huot ME , Laprise P & Mazroui R (2013) Characterization of fragile X mental retardation protein recruitment and dynamics in Drosophila stress granules. PLoS One 8, e55342. 23408971 10.1371/journal.pone.0055342 PMC3567066

[13] Lasko P (2003) Cup‐ling oskar RNA localization and translational control. J Cell Biol 163, 1189–1191. 14691130 10.1083/jcb.200311123 PMC2173710

[14] Lischwe MA , Roberts KD , Yeoman LC & Busch H (1982) Nucleolar specific acidic phosphoprotein C23 is highly methylated. J Biol Chem 257, 14600–14602. 7174653

[15] Plank M , Fischer R , Geoghegan V , Charles PD , Konietzny R , Acuto O , Pears C , Schofield CJ & Kessler BM (2015) Expanding the yeast protein arginine methylome. Proteomics 15, 3232–3243. 26046779 10.1002/pmic.201500032

[16] Chen C , Nott TJ , Jin J & Pawson T (2011) Deciphering arginine methylation: tudor tells the tale. Nat Rev Mol Cell Biol 12, 629–642. 21915143 10.1038/nrm3185

[17] Blanc RS & Richard S (2017) Arginine methylation: the coming of age. Mol Cell 65, 8–24. 28061334 10.1016/j.molcel.2016.11.003

[18] Zhang X & Cheng X (2003) Structure of the predominant protein arginine methyltransferase PRMT1 and analysis of its binding to substrate peptides. Structure 11, 509–520. 12737817 10.1016/s0969-2126(03)00071-6 PMC4030380

[19] Weiss VH , McBride AE , Soriano MA , Filman DJ , Silver PA & Hogle JM (2000) The structure and oligomerization of the yeast arginine methyltransferase, Hmt1. Nat Struct Biol 7, 1165–1171. 11101900 10.1038/82028

[20] Thandapani P , O'Connor TR , Bailey TL & Richard S (2013) Defining the RGG/RG motif. Mol Cell 50, 613–623. 23746349 10.1016/j.molcel.2013.05.021

[21] Hsieh CH , Huang SY , Wu YC , Liu LF , Han CC , Liu YC & Tam MF (2007) Expression of proteins with dimethylarginines in Escherichia coli for protein‐protein interaction studies. Protein Sci 16, 919–928. 17456744 10.1110/ps.062667407 PMC2206645

[22] Frankel A & Clarke S (1999) RNase treatment of yeast and mammalian cell extracts affects *in vitro* substrate methylation by type I protein arginine N‐methyltransferases. Biochem Biophys Res Commun 259, 391–400. 10362520 10.1006/bbrc.1999.0779

[23] Brandariz‐Nunez A , Zeng F , Lam QN & Jin H (2017) Sbp1 modulates the translation of Pab1 mRNA in a poly(A)‐ and RGG‐dependent manner. RNA 24, 43–55. 28986506 10.1261/rna.062547.117 PMC5733569

[24] Nissan T , Rajyaguru P , She M , Song H & Parker R (2010) Decapping activators in *Saccharomyces cerevisiae* act by multiple mechanisms. Mol Cell 39, 773–783. 20832728 10.1016/j.molcel.2010.08.025 PMC2946179

[25] Poornima G , Shah S , Vignesh V , Parker R & Rajyaguru PI (2016) Arginine methylation promotes translation repression activity of eIF4G‐binding protein, Scd6. Nucleic Acids Res 44, 9358–9368. 27613419 10.1093/nar/gkw762 PMC5100564

[26] Buchan JR (2014) mRNP granules. Assembly, function, and connections with disease. RNA Biol 11, 1019–1030. 25531407 10.4161/15476286.2014.972208 PMC4615263

[27] Anderson P & Kedersha N (2009) RNA granules: post‐transcriptional and epigenetic modulators of gene expression. Nat Rev Mol Cell Biol 10, 430–436. 19461665 10.1038/nrm2694

[28] Carroll JS , Munchel SE & Weis K (2011) The DExD/H box ATPase Dhh1 functions in translational repression, mRNA decay, and processing body dynamics. J Cell Biol 194, 527–537. 21844211 10.1083/jcb.201007151 PMC3160580

[29] Lien PT , Izumikawa K , Muroi K , Irie K & Suda Y (2016) Analysis of the physiological activities of Scd6 through its interaction with Hmt1. PLoS One 11, e0164773. 27776129 10.1371/journal.pone.0164773 PMC5077174

[30] Low JK , Hart‐Smith G , Erce MA & Wilkins MR (2013) Analysis of the proteome of *Saccharomyces cerevisiae* for methylarginine. J Proteome Res 12, 3884–3899. 23865587 10.1021/pr400556c

[31] Huang L , Wang Z , Narayanan N & Yang Y (2018) Arginine methylation of the C‐terminus RGG motif promotes TOP3B topoisomerase activity and stress granule localization. Nucleic Acids Res 46, 3061–3074. 29471495 10.1093/nar/gky103 PMC5888246

[32] Poornima G , Mythili R , Nag P , Parbin S , Verma PK , Hussain T & Rajyaguru PI (2019) RGG‐motif self‐association regulates eIF4G‐binding translation repressor protein Scd6. RNA Biol 16, 1215–1227. 31157589 10.1080/15476286.2019.1621623 PMC6693564

