# Supplementary material for: Arginine methylation augments Sbp1 function in translation repression and decapping
Source: FEBS J. 2019 Sep 23;286(23):4693–708. doi: 10.1111/febs.15057 (PMC6916386; doi:10.1111/febs.15057)
Supplement: Supplementary file 1 — Fig. S1. Sbp1 overexpression does not lead to decreased 35S incorporation. Table S1. List of strains used in this study. Table S2. List of plasmids used in this study. [file FEBS-286-4693-s001.zip › febs15057-sup-0001-Supinfo.pdf]

FEBS PRESS
science publishing by scientists
WILEY
The FEBS Journal
www.febsjournal.org

Arginine methylation augments Sbp1 function in translation repression and decapping

Nupur Bhatter, Raju Roy, Shanaya Shah, Sneha P. Sastry, Sabnam Parbin, Rajan Iyappan, Siddharth Kankaria and Purusharth I. Rajyaguru

DOI: 10.1111/febs.15057

Arginine methylation augments Sbp1 function in translation repression and decapping

Nupur Bhatter, Raju Roy, Shanaya Shah[#], Sneha P Sastry, Sabnam Parbin, Rajan Iyappan[$], Siddharth Kankaria and Purusharth I Rajyaguru*

Department of Biochemistry, Indian Institute of Science, C V Raman Road
Bangalore 560012

Sbp1 over expression does not lead to decreased S^{35} incorporation

Supplementary Figure 1: Over expression of Sbp1 does not cause decrease in global protein level as observed by S^{35} incorporation assay. SBP1 gene and its mutants were expressed under galactose-inducible promoter in BG1805 vector backbone and transformed in BY4741 cells. ^{35}S labeled Methionine was added in the media after the cells were shifted to galactose media upon reaching log phase. Time points were taken as indicated in the figure . Cells were lysed by boiling for 5min along with 1X lamellie buffer and loaded on 8% polyacrylamide SDS PAGE gel. After drying the gel, it was exposed to phosphor screen and scanned by phosphorimager.

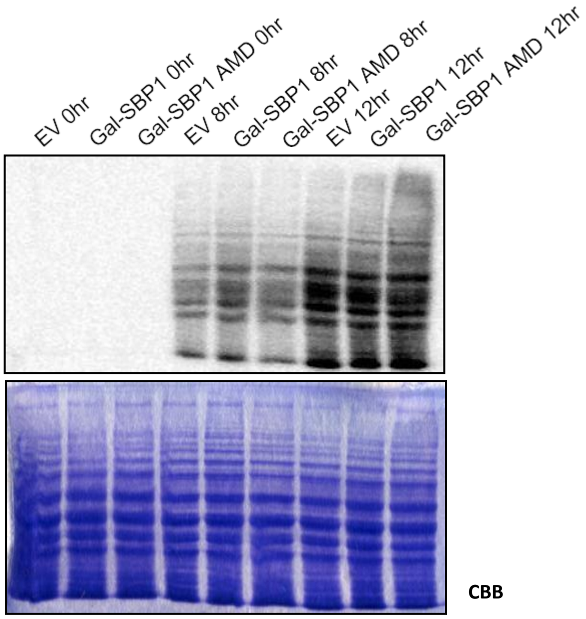

Supplementary Table 1: List of strains

Strains	Genotype	Reference
yPIR1	MATa his3D1 leu2D0 met15D0 ura3D0 ('BY4741')	[24]
yPIR2	MATa his3D1 leu2D0 met15D0 ura3D0 hmt1Δ::KanMX	[24]
yPIR7	MATa his3D1 leu2 ura3 his3 met15 SBP1-GST::KanMX	This study
yPIR8	MATa his3D1 leu2 ura3 his3 met15 SBP1-GST::KanMX hmt1Δ::Hyg	This study
yPIR11	MATa his4-539 leu2-3,112 lys2-201 trp1 ura3-52 dcp1-2::TRP1 ski8Δ::URA3	[8]
yPIR12	MATa leu2 ura3 his3 met15 DHH1-GFP (HIS)	[8]
yPIR3	MATa leu2 ura3 his3 met15 Pab1-GFP (HIS)	[24]
yPIR13	MATa leu2 ura3 his3 met15 Scd6-GFP (HIS)	A kind gift from Dr.Randall Halfmann
yPIR54	MATa leu2 ura3 his3 met15 Scd6-GFP (HIS)hmt1Δ::KanMX	This study

Supplementary Table 2: List of plasmids and constructs

	Description	Vector	Reference
pPIR6	2μ plasmid with gal-inducible promoter and uracil marker used as empty vector control	BG1805	[28]
pPIR14	Vector used for expressing Sbp1 under galactose promoter	BG1805	This study
pPIR15	2μ plasmid expressing Sbp1ΔRGG (Δ 125 to 167 amino acid) under gal-inducible Promoter	BG1805	"
pPIR16	2μ plasmid expressing Sbp1R125A, R129A, R131A, R135A, R137A, R141A, R145A, R149A,R153A, R155A, R159A, R161A and R165A under gal-inducible promoter	BG1805	"
pPIR17	2μ plasmid expressing Sbp1ΔRRM1(37 to 119 aminoacid deleted) under galactose promoter	BG1805	"
pPIR18	2μ plasmid expressing Sbp1ΔRRM2(186 to 274 aminoacid deleted) under galactose promoter	BG1805	"
pPIR20	CEN plamsid with leucine as selectable marker	pRS315	"
pPIR21	CEN plasmid with SBP1 ORF followed by GFP tag with 500 bases upstream and 137 bases downstream using Stu1 as cloning site	pRS315	"
pPIR22	CEN plasmid with SBP1 ΔRGG followed by GFP tag with 500 bases upstream and 137 bases downstream using Stu1 as cloning site	pRS315	"
pPIR23	CEN plasmid with SBP1R125A, R129A, R131A, R135A, R137A, R141A, R145A, R149A, R153A, R155A, R159A, R161A and R165A ORF followed by GFP tag with 500 bases upstream and 137 bases downstream using Stu1 as cloning site	pRS315	"
pPIR24	CEN plasmid with Sbp1 ORF along with 500 bases upstream and 137 bases downstream cloned using Stu1 as cloning site	pRS315	"
pPIR25	CEN plasmid with SBP1 ΔRGG along with 500 bases upstream and 137 bases downstream using Stu1 as cloning site	pRS315	"
pPIR26	CEN plasmid with SBP1R125A, R129A, R131A, R135A, R137A, R141A, R145A, R149A, R153A, R155A, R159A, R161A and R165A ORF followed by GFP tag with 500 bases upstream and 137 bases downstream using Stu1 as cloning site	pRS315	"
pPIR27	Vector with GST-Kanamycin cassette	pFA6a-GST-KanMx6	A kind gift from Dr.Krishnaveni Mishra
pPIR28	Used for expressing Sbp1 with N-terminal 6X His tag and C-terminal FLAG tag cloned using BamH1 and Xho1 restriction site	pPROEX	This study
pPIR33	Used for expressing Sbp1 AMD mutant with N-terminal 6X His tag and C-terminal FLAG tag cloned using BamH1 and Xho1 restriction site	pPROEX	This study
pPIR29	Used for expressing His-Sbp1-FLAG with RGG motif deleted (125 to 167 amino acid)	pPROEX	This study
pPIR30	Used for expressing full length eIF4G1 tagged N-terminus to GST and C-terminus to 6X-HIS	pGEX6P3	[6]
pPIR4	Used for expressing GST	pGEX6P3	[6]

pPIR32	MFA2 cloned under T7 promoter using Sma1 restriction enzyme	pUC18	[24]
pPIR211	2μ plasmid expressing Sbp1R125K, R129K, R131K, R135K, R137K, R141K, R145K, R149K,R153K, R155K, R159K, R161K under gal-inducible promoter	BG1805	This study
